# Outbreak of Ceftriaxone-Resistant *Salmonella enterica* Serovar Typhi, Bangladesh, 2024

**DOI:** 10.3201/eid3107.241987

**Published:** 2025-07

**Authors:** Yogesh Hooda, Arif Mohammad Tanmoy, Sudipta Deb Nath, Anannya Barman Jui, Al Amin, Hafizur Rahman, Neoyman Nasir Shorkar, Naito Kanon, Md Asadur Rahman, Denise O. Garrett, Mohammad Shahidul Islam, Nawshad Uddin Ahmed ASM, Samir K. Saha, Senjuti Saha

**Affiliations:** Child Health Research Foundation, Dhaka, Bangladesh (Y. Hooda, A.M. Tanmoy, S.D. Nath, A.B. Jui, A. Amin, H. Rahman, N.N. Shorkar, N. Kanon, M.S. Islam, A.N.U. Ahmed, S.K. Saha, S. Saha); Popular Diagnostic Centre Limited, Dhaka (M.A. Rahman); Sabin Vaccine Institute, Washington, DC, USA (D.O. Garrett); Bangladesh Shishu Hospital and Institute, Dhaka (S.K. Saha)

**Keywords:** *Salmonella* Typhi, bacteria, antimicrobial resistance, typhoid fever, ceftriaxone resistance, *Salmonella enterica* serovar Typhi, Bangladesh

## Abstract

We report an outbreak of ceftriaxone-resistant *Salmonella enterica* serovar Typhi in Bangladesh; 47 cases were identified during April–September 2024. Isolates belonged to genotype 4.3.1.2 and harbored the *bla*_CTX-M-15_ gene on the pCROB1 plasmid. This genotype-plasmid lineage represents a recent introduction, calling for strengthened surveillance, antimicrobial stewardship, and vaccination strategies.

*Salmonella enterica* serovar Typhi, which causes typhoid fever, remains a major public health concern, particularly in South Asia, which accounts for ≈70% of global cases (*1*). Reports of drug-resistant *Salmonella* Typhi have increased in recent decades. Of particular concern are strains resistant to ceftriaxone, azithromycin, or both (*2*). In 2016, researchers identified an outbreak of extensively drug-resistant *Salmonella* Typhi in Pakistan (*3*), in which strains showed resistance to chloramphenicol, ampicillin, trimethoprim/sulfamethoxazole, fluoroquinolones, and third-generation cephalosporins (*3*). Additional reports noted sporadic cases of independently acquired ceftriaxone-resistant *Salmonella* Typhi from Bangladesh (*4*), India (*5*), and the United Kingdom (*6*). Routine use of ceftriaxone as empirical therapy in South Asia creates selective pressure, heightening the need for public health vigilance to prevent the spread of resistant *Salmonella* Typhi (*2*).

In Bangladesh, *Salmonella* Typhi is the most common cause of bloodstream infections in children >2 months of age; Dhaka, the capital city, is the primary focus of surveillance initiatives (*7*). To broaden the scope of surveillance and monitor the burden of disease and associated antibiotic drug susceptibility patterns nationwide, we began expanding our surveillance network in January 2023 to include clinics affiliated with Popular Diagnostic Centre Ltd (PDCL), a large, Bangladesh-based diagnostic services provider organization. As of May 2024, the network encompassed 20 clinics across 11 districts, including 10 in Dhaka (Figure 1, panel A). Leveraging this expanded passive surveillance for typhoid fever, we report data from January 2023–September 2024, describing the emergence, spread, and genomic epidemiology of a ceftriaxone-resistant clone of *Salmonella* Typhi in Bangladesh. 

**Figure 1:**
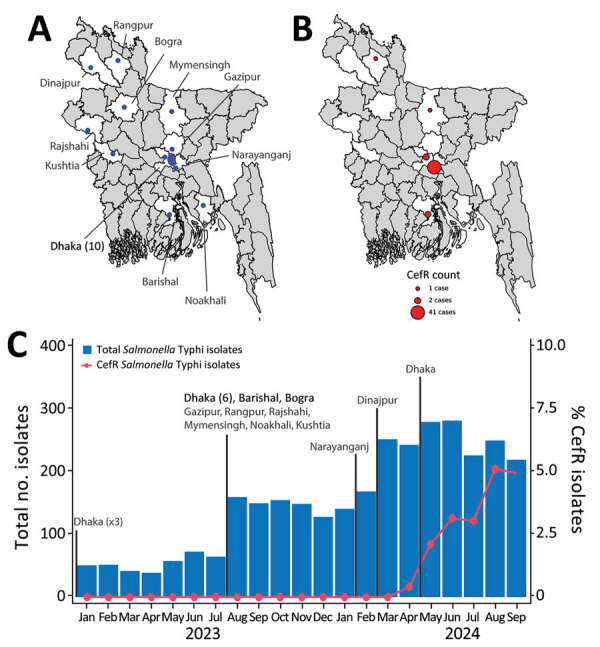
Overview of study of an outbreak of ceftriaxone-resistant *Salmonella* *enterica* serovar Typhi in Bangladesh, 2024. A) Locations of the 20 study clinics across 11 districts of Bangladesh (blue dots). White indicates districts where the study clinics are located; gray indicates districts not included in this study. B) Geographic distribution of CefR *Salmonella* Typhi isolates across Bangladesh. The size of each red dot represents the number of isolates per district. C) Timeline of the surveillance network, showing the inclusion of clinics (text labels) and the total number of *Salmonella* Typhi isolates and CefR isolates detected. Our surveillance efforts began in January 2023 with 3 clinics in Dhaka; more clinics in various locations across Bangladesh were added through May 2024. CefR, ceftriaxone-resistant.

## The Study

PDCL clinics are outpatient facilities that perform physician-prescribed blood cultures for patients. If a blood culture yields *Salmonella* Typhi, technicians transport the isolate to the Child Health Research Foundation (CHRF, https://chrfbd.org) laboratory for serovar confirmation and antibiotic drug susceptibility testing against ampicillin, cefixime, ceftazidime, ceftriaxone, meropenem, chloramphenicol, trimethoprim/sulfamethoxazole, ciprofloxacin, and azithromycin. Those processes follow established methodologies, including biochemical and slide-agglutination tests (*Salmonella*-agglutinating antiserum; Thermo Fisher Scientific, https://www. thermofisher.com) and Clinical and Laboratory Standards Institute–guided Kirby-Bauer disc diffusion methods (Oxoid; Thermo Fisher Scientific) (*8*).

We identified the first ceftriaxone-resistant *Salmonella* Typhi isolate on April 27, 2024, at the PDCL clinic in Narayanganj, a city 30 km from Dhaka, which was added to our surveillance network in February 2024. Subsequent monthly detection of ceftriaxone-resistant strains continued through the study period, culminating in 47 cases by September 2024. Of those, 41 cases were from Narayanganj and 6 were from other districts (Figure 1, panel B). The isolation rate of ceftriaxone-resistant *Salmonella* Typhi increased from 0% to 5% of all isolates during March 2024–September 2024 (Figure 1, panel C). All 47 isolates were resistant to amoxicillin and ceftriaxone, nonsusceptible to fluoroquinolones, but sensitive to chloramphenicol, trimethoprim/sulfamethoxazole, azithromycin, and meropenem (Table).

**Table:** Clinical, demographic, and epidemiologic features of patients with ceftriaxone-resistant *Salmonella* *enterica* serovar Typhi 4.3.1.2.B1 infections based on data from an outbreak in Bangladesh, 2024

Characteristic	No. (%) patients, n = 21
Sex	
M	15 (72)
F	6 (28)
Age, y	
1–5	7 (33)
6–10	3 (14)
11–15	4 (19)
16–20	3 (14)
21–25	2 (10)
>25	2 (10)
Clinical characteristics	
Fever	21 (100)
Headache	11 (52)
Cough	10 (47)
Abdominal pain	11 (52)
Breathing difficulty	3 (14)
Vomiting	7 (33)
Diarrhea	6 (28)
Jaundice*	1 (5)
Nausea	2 (10)
Anorexia	2 (10)
Treatment outcome	
Recovered	21 (100)
Treatment location	
Outpatient department	14 (67)
Inpatient department	7 (33)
Initial antibiotic received	
Third-generation cephalosporin	12 (57)
Cefixime	7 (30)
Ceftriaxone	4 (19)
Ceftibuten	1 (5)
Second-generation cephalosporin: cefuroxime	1 (5)
Azithromycin	4 (19)
Ciprofloxacin	1 (5)
Ciprofloxacin + azithromycin	1 (5)
Tazobactam/piperacillin	1 (5)
Unknown	1 (5)
Second antibiotic received	
Third-generation cephalosporin: ceftriaxone	2 (10)
Meropenem	5 (24)
Azithromycin	1 (5)
Amoxicillin/clavulanic acid, amikacin	1 (5)
Trimethoprim/sulfamethoxazole	1 (5)
Unknown	1 (5)

*Participant had concomitant hepatitis A virus infection.

To investigate the basis of ceftriaxone resistance, we performed whole-genome sequencing on 17 of the 47 resistant *Salmonella* Typhi isolates (Appendix). Those isolates included 14 from Narayanganj and 1 each from Barishal, Mymensingh, and Rangpur. We prepared genomic libraries using the New England Biolabs DNA library preparation kit (New England Biolabs, https://www.neb.com) and sequenced on a NextSeq2000 platform (2 × 150 bp). We performed genome assembly using Unicycler v0.5.1 (*9*) and analyzed raw fastq files with Mykrobe v0.13.0 (D.J. Ingle et al., unpub. data, https://www.biorxiv.org/content/10.1101/2024.09.30.613582v1). 

We classified all 17 ceftriaxone-resistant genomes as genotype 4.3.1.2, noting that ceftriaxone resistance was conferred by the *bla*_CTX-M-15_ gene, which showed 100% sequence identity with the gene found in extensively drug-resistant *Salmonella* Typhi strains from Pakistan (genotype 4.3.1.1.P1) (*3*). We performed phylogenetic analysis using Bowtie2, Samtools, Gubbins, and RAxML, following the pipeline described previously (*10*).

**Figure 2:**
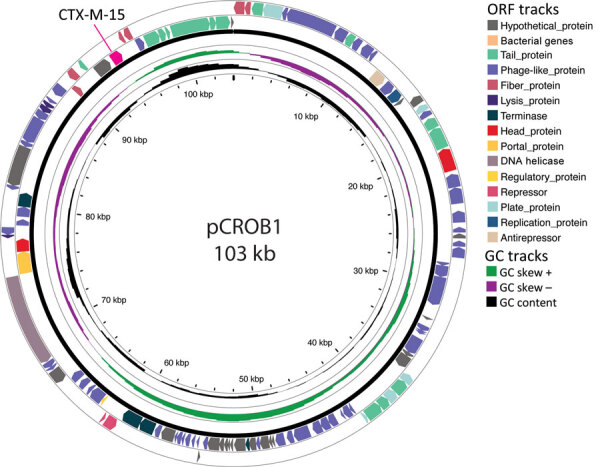
Annotated map of plasmid pCROB1 from study of an outbreak of ceftriaxone-resistant *Salmonella enterica* serovar Typhi, Bangladesh, 2024. pCROB1 harbors the *bla*_CTX-M-15_ gene and includes an intact phage element, as annotated using PHASTEST (*12*). Tracks around the plasmid map highlight GC content and predicted ORFs. GC, guanine and cytosine nucleotides; OFR, open reading frame.

We identified an IncY plasmid in all 17 sequenced strains, which we further characterized using plasmidSPAdes v3.15.5 (*11*). We generated the longest plasmid sequence of 103.9 kbp from isolate STY_0313. This IncY plasmid, referred to as pCROB1, carried the *bla*_CTX-M-15_ gene and encoded a phage element (Figure 2). Public Health England previously identified a similar phage-plasmid associated with genotype 4.3.1.1 among travel-related typhoid cases from Iraq (*6*), suggesting a unique genotype-plasmid lineage underpinning the ongoing outbreak in Bangladesh.

**Figure 3:**
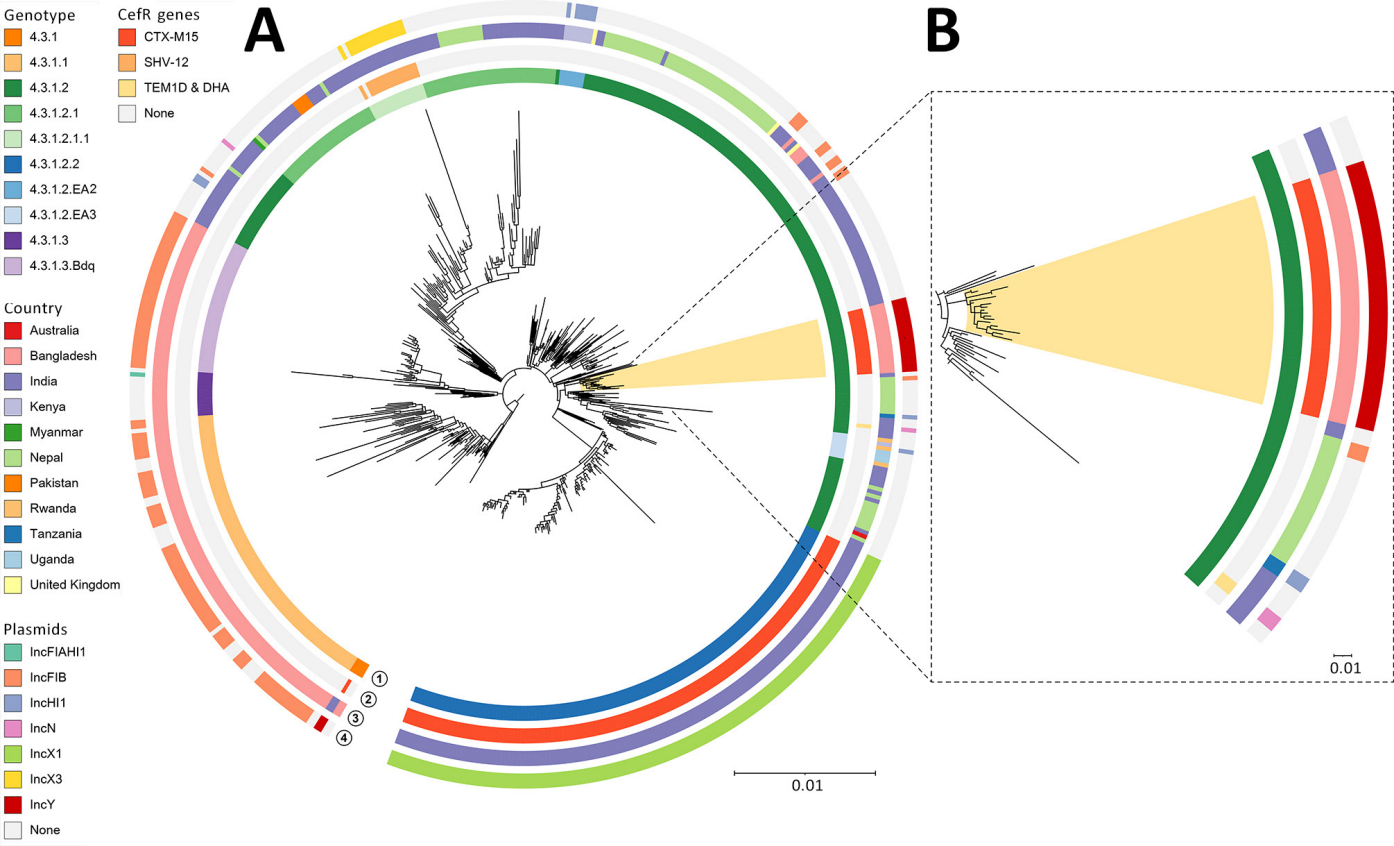
Phylogenetic tree of *Salmonella* Typhi genotype 4.3.1, including the 17 CefR 4.3.1.2.B1 strains detected in findings from a study of an outbreak of CefR *S. enterica* serovar Typhi, Bangladesh, 2024. A) Phylogenetic tree of 546 genomes belonging to genotype 4.3.1 and its subtypes. CefR strains sequenced in our study belong to genotype 4.3.1 and are highlighted in yellow. Tree was built following a pipeline described earlier (*10*) and displays different CefR genes, countries of isolation, and associated plasmid elements. For context, 529 genomes from genotype 4.3.1 and its subtypes were also included. Of those, 249 (10%) were randomly selected from 2,567 genomes (genotype 4.3.1.2 and subtypes) available on Pathogenwatch by genotype, year, and country (accessions available for 2,542; accessed on 14 July 2024), and 280 were from previous studies conducted in Bangladesh, India, and Pakistan (*14*). B) Zoomed-in view of the subclade containing the CefR strains from our study in Bangladesh. Scale bars indicate mean nucleotide substitutions per site. CefR, ceftriaxone-resistant.

Genotype 4.3.1.2 is rarely identified in Bangladesh and accounted for only 0.5% (7/1,356) of all *Salmonella* Typhi whole-genome sequences available for 1999–2018 (*13*). The genotype is more commonly found in India and Nepal (Figure 3). To investigate the phylogenetic context of genotype 4.3.1.2, we constructed a tree using global database sequences from genotype 4.3.1 and its subtypes, with a focus on 4.3.1.2 and its sublineages (Appendix). We found that the recent ceftriaxone resistant isolates from Bangladesh are closely related to strains from India and Nepal, rather than to earlier 4.3.1.2 genotypes from Bangladesh (Figure 3). However, our research showed no strains from India or Nepal were reported to be ceftriaxone-resistant or to carry the pCROB1 plasmid. We propose naming this distinct subclade lineage 4.3.1.2.B1. Although researchers have reported ceftriaxone resistance in genotype 4.3.1.2 strains from India (*5*), the molecular basis of resistance in lineage 4.3.1.2.B1 differs, suggesting an independent acquisition of the ceftriaxone-encoding phage-plasmid by this genotype.

To better understand the progression of disease in patients with ceftriaxone-resistant typhoid, we selected 35 cases (identified through August 31, 2024) for telephone interviews, successfully completing 21 (60%). Most (65%, 13/21) patients initially received either second-generation (cefuroxime 5%, 1/21) or third-generation (57%, 12/21; including cefixime [30%, 7/21], ceftriaxone [19%, 4/21], and ceftibuten [5%, 1/21]) cephalosporins. Forty-six percent (6/13) of patients switched to meropenem (23%, 3/13), azithromycin (8%, 1/13), or trimethoprim/sulfamethoxazole (8%, 1/13). One patient could not recall the second antibiotic used. All patients recovered; average illness duration was 19 (range 12–28) days. No patients reported travel outside Bangladesh, suggesting local circulation of this strain. Three cases occurred outside Narayanganj, the initial outbreak site; 2 patients had no travel history to Narayanganj within 15 days of illness onset, indicating potential spread to other districts.

## Conclusions

Our detection of a unique genotype-plasmid lineage—4.3.1.2.B1, carrying the *bla*_CTX-M-15_ gene on the pCROB1 plasmid—associated with an outbreak of ceftriaxone-resistant *Salmonella* Typhi in Bangladesh represents a concerning development because of the strain’s potential for regional and international spread (*2*). Given the observed local spread of this lineage, healthcare systems may need to prepare for a shift back to older antibiotics, such as trimethoprim/sulfamethoxazole and chloramphenicol. Of note, 4.3.1.2.B1 strains remain sensitive to those drugs, indicating that first-line antibiotic drugs may serve as viable alternatives to azithromycin and meropenem. Seven patients in this study received third-generation cephalosporins and recovered from fever; however, we did not follow those cases to investigate relapse rates and other complications that could result from improper antibiotic use.

Our investigation of a unique strain of ceftriaxone-resistant *Salmonella* Typhi emerging in Bangladesh underscores the role of widespread ceftriaxone use in selecting for antimicrobial-resistant strains of typhoid fever. The outbreak was detected in April 2024, but the clone could have been circulating in Narayanganj before detection in our surveillance network. Because ceftriaxone remains a cornerstone of empirical treatment for typhoid, its declining efficacy is of great public health concern. Public health efforts should focus on bolstering antimicrobial stewardship and public education on appropriate antibiotic drug use, strengthening surveillance systems, and implementing and promoting immunization with typhoid conjugate vaccines to curb further spread of resistant strains.

Appendix Additional information for outbreak of ceftriaxone-resistant *Salmonella enterica* serovar Typhi, Bangladesh, 2024.
