# Supplementary material for: Outbreak of Ceftriaxone-Resistant Salmonella enterica Serovar Typhi, Bangladesh, 2024
Source: Emerg Infect Dis. 2025 Jul;31(7):1460–5. doi: 10.3201/eid3107.241987 (PMC12205446; doi:10.3201/eid3107.241987)
Supplement: Appendix — Additional information for outbreak of ceftriaxone-resistant Salmonella enterica serovar Typhi, Bangladesh, 2024. [file 24-1987-Techapp-s1.pdf]

Article DOI: http://doi.org/10.3201/eid3107.241987

EID cannot ensure accessibility for supplementary materials supplied by authors. Readers who have difficulty accessing supplementary content should contact the authors for assistance.

Outbreak of Ceftriaxone-Resistant *Salmonella enterica* Serovar Typhi, Bangladesh, 2024

Appendix

Appendix Table. List of genomes used for phylogenetic tree analysis from a study of an outbreak of ceftriaxone-resistant *Salmonella enterica* serovar Typhi, Bangladesh, 2024

.

Accession	Genotype	Country	Study	Format	Ceftriaxone_res	Plasmids
DRR071021	4.3.1.2	India	Pathogenwatch	fastq	S	none
DRR071031	4.3.1.2	India	Pathogenwatch	fastq	S	none
DRR071055	4.3.1.2.1	India	Pathogenwatch	fastq	S	none
DRR071067	4.3.1.2.1	Nepal	Pathogenwatch	fastq	S	none
ERR1017094	4.3.1.2	India	Pathogenwatch	fastq	S	none
ERR1079231	4.3.1.2	Nepal	Pathogenwatch	fastq	S	none
ERR1079260	4.3.1.2	Nepal	Pathogenwatch	fastq	S	none
ERR1079263	4.3.1.2.1	Nepal	Pathogenwatch	fastq	S	none
ERR1079272	4.3.1.2	Nepal	Pathogenwatch	fastq	S	none
ERR1079294	4.3.1.2.1	Nepal	Pathogenwatch	fastq	S	none
ERR1079302	4.3.1.2.1	Nepal	Pathogenwatch	fastq	S	none
ERR1079306	4.3.1.2	Nepal	Pathogenwatch	fastq	S	none
ERR108685	4.3.1.2	Tanzania	Pathogenwatch	fastq	S	IncHI1
ERR11458673	4.3.1.2.2	India	Thirumoorthy_et_al	fastq	R: blaCTX-M-15	IncX1
ERR11458675	4.3.1.2.2	India	Thirumoorthy_et_al	fastq	R: blaCTX-M-15	IncX1
ERR11458679	4.3.1.2.2	India	Thirumoorthy_et_al	fastq	R: blaCTX-M-15	IncX1
ERR11458680	4.3.1.2.2	India	Thirumoorthy_et_al	fastq	R: blaCTX-M-15	IncX1
ERR11458681	4.3.1.2.2	India	Thirumoorthy_et_al	fastq	R: blaCTX-M-15	IncX1
ERR11458682	4.3.1.2.2	India	Thirumoorthy_et_al	fastq	R: blaCTX-M-15	IncX1
ERR12081789	4.3.1.2.2	India	Thirumoorthy_et_al	fastq	R: blaCTX-M-15	IncX1
ERR12081790	4.3.1.2.2	India	Thirumoorthy_et_al	fastq	R: blaCTX-M-15	IncX1
ERR12081791	4.3.1.2.2	India	Thirumoorthy_et_al	fastq	R: blaCTX-M-15	IncX1
ERR12081792	4.3.1.2.2	India	Thirumoorthy_et_al	fastq	R: blaCTX-M-15	IncX1
ERR12081793	4.3.1.2.2	India	Thirumoorthy_et_al	fastq	R: blaCTX-M-15	IncX1
ERR12081794	4.3.1.2.2	India	Thirumoorthy_et_al	fastq	R: blaCTX-M-15	IncX1
ERR12081795	4.3.1.2.2	India	Thirumoorthy_et_al	fastq	R: blaCTX-M-15	IncX1
ERR12081796	4.3.1.2.2	India	Thirumoorthy_et_al	fastq	R: blaCTX-M-15	IncX1
ERR12081797	4.3.1.2.2	India	Thirumoorthy_et_al	fastq	R: blaCTX-M-15	IncX1
ERR12081798	4.3.1.2.2	India	Thirumoorthy_et_al	fastq	R: blaCTX-M-15	IncX1
ERR12081799	4.3.1.2.2	India	Thirumoorthy_et_al	fastq	R: blaCTX-M-15	IncX1
ERR12081800	4.3.1.2.2	India	Thirumoorthy_et_al	fastq	R: blaCTX-M-15	IncX1
ERR12081801	4.3.1.2.2	India	Thirumoorthy_et_al	fastq	R: blaCTX-M-15	IncX1
DRR071021	4.3.1.2	India	Pathogenwatch	fastq	S	none
DRR071031	4.3.1.2	India	Pathogenwatch	fastq	S	none
DRR071055	4.3.1.2.1	India	Pathogenwatch	fastq	S	none
DRR071067	4.3.1.2.1	Nepal	Pathogenwatch	fastq	S	none
ERR1017094	4.3.1.2	India	Pathogenwatch	fastq	S	none
ERR1079231	4.3.1.2	Nepal	Pathogenwatch	fastq	S	none
ERR1079260	4.3.1.2	Nepal	Pathogenwatch	fastq	S	none
ERR1079263	4.3.1.2.1	Nepal	Pathogenwatch	fastq	S	none
ERR1079272	4.3.1.2	Nepal	Pathogenwatch	fastq	S	none
ERR1079294	4.3.1.2.1	Nepal	Pathogenwatch	fastq	S	none
ERR1079302	4.3.1.2.1	Nepal	Pathogenwatch	fastq	S	none

Accession	Genotype	Country	Study	Format	Ceftriaxone_res	Plasmids
ERR1079306	4.3.1.2	Nepal	Pathogenwatch	fastq	S	none
ERR108685	4.3.1.2	Tanzania	Pathogenwatch	fastq	S	IncHI1
ERR11458673	4.3.1.2.2	India	Thirumoorthy_et_al	fastq	R: blaCTX-M-15	IncX1
ERR11458675	4.3.1.2.2	India	Thirumoorthy_et_al	fastq	R: blaCTX-M-15	IncX1
ERR11458679	4.3.1.2.2	India	Thirumoorthy_et_al	fastq	R: blaCTX-M-15	IncX1
ERR11458680	4.3.1.2.2	India	Thirumoorthy_et_al	fastq	R: blaCTX-M-15	IncX1
ERR11458681	4.3.1.2.2	India	Thirumoorthy_et_al	fastq	R: blaCTX-M-15	IncX1
ERR11458682	4.3.1.2.2	India	Thirumoorthy_et_al	fastq	R: blaCTX-M-15	IncX1
ERR12081789	4.3.1.2.2	India	Thirumoorthy_et_al	fastq	R: blaCTX-M-15	IncX1
ERR12081790	4.3.1.2.2	India	Thirumoorthy_et_al	fastq	R: blaCTX-M-15	IncX1
ERR12081791	4.3.1.2.2	India	Thirumoorthy_et_al	fastq	R: blaCTX-M-15	IncX1
ERR12081792	4.3.1.2.2	India	Thirumoorthy_et_al	fastq	R: blaCTX-M-15	IncX1
ERR12081793	4.3.1.2.2	India	Thirumoorthy_et_al	fastq	R: blaCTX-M-15	IncX1
ERR12081794	4.3.1.2.2	India	Thirumoorthy_et_al	fastq	R: blaCTX-M-15	IncX1
ERR12081795	4.3.1.2.2	India	Thirumoorthy_et_al	fastq	R: blaCTX-M-15	IncX1
ERR12081796	4.3.1.2.2	India	Thirumoorthy_et_al	fastq	R: blaCTX-M-15	IncX1
ERR12081797	4.3.1.2.2	India	Thirumoorthy_et_al	fastq	R: blaCTX-M-15	IncX1
ERR12081798	4.3.1.2.2	India	Thirumoorthy_et_al	fastq	R: blaCTX-M-15	IncX1
ERR12081799	4.3.1.2.2	India	Thirumoorthy_et_al	fastq	R: blaCTX-M-15	IncX1
ERR12081800	4.3.1.2.2	India	Thirumoorthy_et_al	fastq	R: blaCTX-M-15	IncX1
ERR12081801	4.3.1.2.2	India	Thirumoorthy_et_al	fastq	R: blaCTX-M-15	IncX1
ERR12081844	4.3.1.2.2	India	Thirumoorthy_et_al	fastq	R: blaCTX-M-15	IncX1
ERR12081845	4.3.1.2.2	India	Thirumoorthy_et_al	fastq	R: blaCTX-M-15	IncX1
ERR12081846	4.3.1.2.2	India	Thirumoorthy_et_al	fastq	R: blaCTX-M-15	IncX1
ERR12081847	4.3.1.2.2	India	Thirumoorthy_et_al	fastq	R: blaCTX-M-15	IncX1
ERR12081848	4.3.1.2.2	India	Thirumoorthy_et_al	fastq	R: blaCTX-M-15	IncX1
ERR12081849	4.3.1.2.2	India	Thirumoorthy_et_al	fastq	R: blaCTX-M-15	IncX1
ERR12081850	4.3.1.2.2	India	Thirumoorthy_et_al	fastq	R: blaCTX-M-15	IncX1
ERR12081851	4.3.1.2.2	India	Thirumoorthy_et_al	fastq	R: blaCTX-M-15	IncX1
ERR12081852	4.3.1.2.2	India	Thirumoorthy_et_al	fastq	R: blaCTX-M-15	IncX1
ERR12081853	4.3.1.2.2	India	Thirumoorthy_et_al	fastq	R: blaCTX-M-15	IncX1
ERR12081854	4.3.1.2.2	India	Thirumoorthy_et_al	fastq	R: blaCTX-M-15	IncX1
ERR12081855	4.3.1.2.2	India	Thirumoorthy_et_al	fastq	R: blaCTX-M-15	IncX1
ERR12081868	4.3.1.2.2	India	Thirumoorthy_et_al	fastq	R: blaCTX-M-15	IncX1
ERR12081869	4.3.1.2.2	India	Thirumoorthy_et_al	fastq	R: blaCTX-M-15	IncX1
ERR12081870	4.3.1.2.2	India	Thirumoorthy_et_al	fastq	R: blaCTX-M-15	IncX1
ERR12081872	4.3.1.2.2	India	Thirumoorthy_et_al	fastq	R: blaCTX-M-15	IncX1
ERR12081873	4.3.1.2.2	India	Thirumoorthy_et_al	fastq	R: blaCTX-M-15	IncX1
ERR12081874	4.3.1.2.2	India	Thirumoorthy_et_al	fastq	R: blaCTX-M-15	IncX1
ERR12081875	4.3.1.2.2	India	Thirumoorthy_et_al	fastq	R: blaCTX-M-15	IncX1
ERR12081876	4.3.1.2.2	India	Thirumoorthy_et_al	fastq	R: blaCTX-M-15	IncX1
ERR12081877	4.3.1.2.2	India	Thirumoorthy_et_al	fastq	R: blaCTX-M-15	IncX1
ERR12081878	4.3.1.2.2	India	Thirumoorthy_et_al	fastq	R: blaCTX-M-15	IncX1
ERR12081879	4.3.1.2.2	India	Thirumoorthy_et_al	fastq	R: blaCTX-M-15	IncX1
ERR12081880	4.3.1.2.2	India	Thirumoorthy_et_al	fastq	R: blaCTX-M-15	IncX1
ERR12081882	4.3.1.2.2	India	Thirumoorthy_et_al	fastq	R: blaCTX-M-15	IncX1
ERR12081883	4.3.1.2.2	India	Thirumoorthy_et_al	fastq	R: blaCTX-M-15	IncX1
ERR12081884	4.3.1.2.2	India	Thirumoorthy_et_al	fastq	R: blaCTX-M-15	IncX1
ERR12081885	4.3.1.2.2	India	Thirumoorthy_et_al	fastq	R: blaCTX-M-15	IncX1
ERR12081886	4.3.1.2.2	India	Thirumoorthy_et_al	fastq	R: blaCTX-M-15	IncX1
ERR12081887	4.3.1.2.2	India	Thirumoorthy_et_al	fastq	R: blaCTX-M-15	IncX1
ERR12081888	4.3.1.2.2	India	Thirumoorthy_et_al	fastq	R: blaCTX-M-15	IncX1
ERR12081889	4.3.1.2.2	India	Thirumoorthy_et_al	fastq	R: blaCTX-M-15	IncX1
ERR12081890	4.3.1.2.2	India	Thirumoorthy_et_al	fastq	R: blaCTX-M-15	IncX1
ERR12081891	4.3.1.2.2	India	Thirumoorthy_et_al	fastq	R: blaCTX-M-15	IncX1
ERR12081892	4.3.1.2.2	India	Thirumoorthy_et_al	fastq	R: blaCTX-M-15	IncX1
ERR12081893	4.3.1.2.2	India	Thirumoorthy_et_al	fastq	R: blaCTX-M-15	IncX1
ERR12081894	4.3.1.2.2	India	Thirumoorthy_et_al	fastq	R: blaCTX-M-15	IncX1
ERR12081895	4.3.1.2.2	India	Thirumoorthy_et_al	fastq	R: blaCTX-M-15	IncX1
ERR12081896	4.3.1.2.2	India	Thirumoorthy_et_al	fastq	R: blaCTX-M-15	IncX1
ERR12081897	4.3.1.2.2	India	Thirumoorthy_et_al	fastq	R: blaCTX-M-15	IncX1
ERR12081898	4.3.1.2.2	India	Thirumoorthy_et_al	fastq	R: blaCTX-M-15	IncX1
ERR12081899	4.3.1.2.2	India	Thirumoorthy_et_al	fastq	R: blaCTX-M-15	IncX1
ERR12081900	4.3.1.2.2	India	Thirumoorthy_et_al	fastq	R: blaCTX-M-15	IncX1
ERR12081901	4.3.1.2.2	India	Thirumoorthy_et_al	fastq	R: blaCTX-M-15	IncX1

Accession	Genotype	Country	Study	Format	Ceftriaxone_res	Plasmids
ERR12081902	4.3.1.2.2	India	Thirumoorthy_et_al	fastq	R: blaCTX-M-15	IncX1
ERR12081903	4.3.1.2.2	India	Thirumoorthy_et_al	fastq	R: blaCTX-M-15	IncX1
ERR12081904	4.3.1.2.2	India	Thirumoorthy_et_al	fastq	R: blaCTX-M-15	IncX1
ERR12081905	4.3.1.2.2	India	Thirumoorthy_et_al	fastq	R: blaCTX-M-15	IncX1
ERR12081907	4.3.1.2.2	India	Thirumoorthy_et_al	fastq	R: blaCTX-M-15	IncX1
ERR12081908	4.3.1.2.2	India	Thirumoorthy_et_al	fastq	R: blaCTX-M-15	IncX1
ERR12081909	4.3.1.2.2	India	Thirumoorthy_et_al	fastq	R: blaCTX-M-15	IncX1
ERR12081910	4.3.1.2.2	India	Thirumoorthy_et_al	fastq	R: blaCTX-M-15	IncX1
ERR12081911	4.3.1.2.2	India	Thirumoorthy_et_al	fastq	R: blaCTX-M-15	IncX1
ERR12081912	4.3.1.2.2	India	Thirumoorthy_et_al	fastq	R: blaCTX-M-15	IncX1
ERR12081913	4.3.1.2.2	India	Thirumoorthy_et_al	fastq	R: blaCTX-M-15	IncX1
ERR12081914	4.3.1.2.2	India	Thirumoorthy_et_al	fastq	R: blaCTX-M-15	IncX1
ERR12081921	4.3.1.2.2	India	Thirumoorthy_et_al	fastq	R: blaCTX-M-15	IncX1
ERR12081923	4.3.1.2.2	India	Thirumoorthy_et_al	fastq	R: blaCTX-M-15	IncX1
ERR12081924	4.3.1.2.2	India	Thirumoorthy_et_al	fastq	R: blaCTX-M-15	IncX1
ERR12081925	4.3.1.2.2	India	Thirumoorthy_et_al	fastq	R: blaCTX-M-15	IncX1
ERR12081926	4.3.1.2.2	India	Thirumoorthy_et_al	fastq	R: blaCTX-M-15	IncX1
ERR12081927	4.3.1.2.2	India	Thirumoorthy_et_al	fastq	R: blaCTX-M-15	IncX1
ERR12081928	4.3.1.2.2	India	Thirumoorthy_et_al	fastq	R: blaCTX-M-15	IncX1
ERR12081929	4.3.1.2.2	India	Thirumoorthy_et_al	fastq	R: blaCTX-M-15	IncX1
ERR12652108	4.3.1.2.2	India	Thirumoorthy_et_al	fastq	R: blaCTX-M-15	IncX1
ERR12652110	4.3.1.2.2	India	Thirumoorthy_et_al	fastq	R: blaCTX-M-15	IncX1
ERR12652111	4.3.1.2.2	India	Thirumoorthy_et_al	fastq	R: blaCTX-M-15	IncX1
ERR12652112	4.3.1.2.2	India	Thirumoorthy_et_al	fastq	R: blaCTX-M-15	IncX1
ERR12652113	4.3.1.2.2	India	Thirumoorthy_et_al	fastq	R: blaCTX-M-15	IncX1
ERR12652114	4.3.1.2.2	India	Thirumoorthy_et_al	fastq	R: blaCTX-M-15	IncX1
ERR12652115	4.3.1.2.2	India	Thirumoorthy_et_al	fastq	R: blaCTX-M-15	IncX1
ERR12652116	4.3.1.2.2	India	Thirumoorthy_et_al	fastq	R: blaCTX-M-15	IncX1
ERR12652117	4.3.1.2.2	India	Thirumoorthy_et_al	fastq	R: blaCTX-M-15	IncX1
ERR1556056	4.3.1.2.EA3	Uganda	Pathogenwatch	fastq	S	none
ERR1556080	4.3.1.2.EA3	Uganda	Pathogenwatch	fastq	S	none
ERR1788758	4.3.1.2	India	Pathogenwatch	fastq	S	none
ERR1788869	4.3.1.2	India	Pathogenwatch	fastq	S	none
ERR1788876	4.3.1.2	India	Pathogenwatch	fastq	S	none
ERR1788880	4.3.1.2	India	Pathogenwatch	fastq	S	none
ERR1788895	4.3.1.2.1	India	Pathogenwatch	fastq	S	none
ERR1788906	4.3.1.2	India	Pathogenwatch	fastq	S	none
ERR1788926	4.3.1.2.1	India	Pathogenwatch	fastq	S	none
ERR1837135	4.3.1.2	Nepal	Pathogenwatch	fastq	S	none
ERR1837148	4.3.1.2	Nepal	Pathogenwatch	fastq	S	none
ERR1837175	4.3.1.2	Nepal	Pathogenwatch	fastq	S	none
ERR1837184	4.3.1.2	Nepal	Pathogenwatch	fastq	S	none
ERR1837236	4.3.1.2	Nepal	Pathogenwatch	fastq	S	none
ERR1837252	4.3.1.2	Nepal	Pathogenwatch	fastq	S	none
ERR1837265	4.3.1.2	Nepal	Pathogenwatch	fastq	S	IncFIB
ERR1837267	4.3.1.2	Nepal	Pathogenwatch	fastq	S	none
ERR2213835	4.3.1.2	Nepal	Pathogenwatch	fastq	S	none
ERR2213852	4.3.1.2	Nepal	Pathogenwatch	fastq	S	none
ERR2213860	4.3.1.2	Nepal	Pathogenwatch	fastq	S	none
ERR2213864	4.3.1.2	Nepal	Pathogenwatch	fastq	S	none
ERR2213867	4.3.1.2	Nepal	Pathogenwatch	fastq	S	none
ERR2525484	4.3.1.2.EA2	Kenya	Pathogenwatch	fastq	S	IncHI1
ERR279346	4.3.1.2	India	Pathogenwatch	fastq	S	IncHI1
ERR2909543	4.3.1.2.EA2	Kenya	Pathogenwatch	fastq	S	IncHI1
ERR2909580	4.3.1.2.EA2	Kenya	Pathogenwatch	fastq	S	IncHI1
ERR3332584	4.3.1.2.EA2	Kenya	Pathogenwatch	fastq	S	IncHI1
ERR3332591	4.3.1.2.EA3	Kenya	Pathogenwatch	fastq	S	none
ERR3332660	4.3.1.2.EA2	Kenya	Pathogenwatch	fastq	S	none
ERR3332709	4.3.1.2.EA2	Kenya	Pathogenwatch	fastq	S	IncHI1
ERR338138	4.3.1.2.EA2	Kenya	Pathogenwatch	fastq	S	IncHI1
ERR343262	4.3.1.2	India	Pathogenwatch	fastq	S	IncFIB
ERR343271	4.3.1.2	India	Pathogenwatch	fastq	S	IncFIB
ERR343320	4.3.1.2	India	Pathogenwatch	fastq	S	none
ERR343323	4.3.1.2	India	Pathogenwatch	fastq	S	none
ERR352271	4.3.1.2	India	Pathogenwatch	fastq	S	none

Accession	Genotype	Country	Study	Format	Ceftriaxone_res	Plasmids
ERR352276	4.3.1.2	Australia	Pathogenwatch	fastq	S	none
ERR352284	4.3.1.2.1	India	Pathogenwatch	fastq	S	none
ERR352338	4.3.1.2	Nepal	Pathogenwatch	fastq	S	none
ERR3527963	4.3.1	India	Sah_et_al	fastq	R: blaCTX-M-15	IncY
ERR3527964	4.3.1	India	Sah_et_al	fastq	S	IncY
ERR357594	4.3.1.2	Nepal	Pathogenwatch	fastq	S	none
ERR357620	4.3.1.2	Nepal	Pathogenwatch	fastq	S	none
ERR357834	4.3.1.2.1	India	Pathogenwatch	fastq	S	none
ERR3804505	4.3.1.2.1	India	Pathogenwatch	fastq	S	none
ERR3804512	4.3.1.2.1	India	Pathogenwatch	fastq	S	none
ERR3804545	4.3.1.2	India	Pathogenwatch	fastq	S	none
ERR3804581	4.3.1.2	India	Pathogenwatch	fastq	S	none
ERR3804584	4.3.1.2.1	India	Pathogenwatch	fastq	S	none
ERR3804590	4.3.1.2	India	Pathogenwatch	fastq	S	none
ERR4025159	4.3.1.2.1	Nepal	Pathogenwatch	fastq	S	none
ERR4025387	4.3.1.2.1	Pakistan	Pathogenwatch	fastq	S	none
ERR4133922	4.3.1.2	Nepal	Pathogenwatch	fastq	S	none
ERR4133939	4.3.1.2	Nepal	Pathogenwatch	fastq	S	none
ERR4133943	4.3.1.2	Nepal	Pathogenwatch	fastq	S	none
ERR4133987	4.3.1.2	Nepal	Pathogenwatch	fastq	S	none
ERR4134001	4.3.1.2	Nepal	Pathogenwatch	fastq	S	none
ERR4134004	4.3.1.2	Nepal	Pathogenwatch	fastq	S	none
ERR420414	4.3.1.2	India	Pathogenwatch	fastq	S	IncHI1
ERR4634491	4.3.1.2	India	Pathogenwatch	fastq	S	none
ERR4634511	4.3.1.2	India	Pathogenwatch	fastq	S	none
ERR4634514	4.3.1.2	India	Pathogenwatch	fastq	S	none
ERR4634518	4.3.1.2	India	Pathogenwatch	fastq	S	none
ERR4634524	4.3.1.2	India	Pathogenwatch	fastq	S	none
ERR4634526	4.3.1.2	India	Pathogenwatch	fastq	S	none
ERR4634539	4.3.1.2	India	Pathogenwatch	fastq	S	none
ERR4634540	4.3.1.2	India	Pathogenwatch	fastq	S	none
ERR4634555	4.3.1.2	India	Pathogenwatch	fastq	S	none
ERR4634557	4.3.1.2	India	Pathogenwatch	fastq	S	none
ERR4634569	4.3.1.2	India	Pathogenwatch	fastq	S	IncFIB
ERR4634580	4.3.1.2	India	Pathogenwatch	fastq	S	none
ERR4634582	4.3.1.2	India	Pathogenwatch	fastq	S	none
ERR4635595	4.3.1.2.1.1	India	Argimon_et_al	fastq	R: blaSHV-12	IncX3
ERR4635768	4.3.1.2.1.1	India	Argimon_et_al	fastq	R: blaSHV-12	IncX3
ERR4635777	4.3.1.2	India	Pathogenwatch	fastq	S	none
ERR4635780	4.3.1.2.1	India	Pathogenwatch	fastq	S	none
ERR4635787	4.3.1.2.1.1	India	Argimon_et_al	fastq	R: blaSHV-12	IncX3
ERR4635805	4.3.1.2.1.1	India	Argimon_et_al	fastq	R: blaSHV-12	IncX3
ERR4635808	4.3.1.2.1.1	India	Argimon_et_al	fastq	R: blaSHV-12	IncX3
ERR4635820	4.3.1.2.1.1	India	Argimon_et_al	fastq	R: blaSHV-12	IncX3
ERR4635821	4.3.1.2.1.1	India	Argimon_et_al	fastq	R: blaSHV-12	IncX3
ERR4635839	4.3.1.2.1.1	India	Argimon_et_al	fastq	R: blaSHV-12	IncX3
ERR4635842	4.3.1.2.1	India	Pathogenwatch	fastq	S	none
ERR4635845	4.3.1.2.1	India	Pathogenwatch	fastq	S	none
ERR4635849	4.3.1.2.1.1	India	Argimon_et_al	fastq	R: blaSHV-12	IncX3
ERR4695904	4.3.1.2.1	India	Pathogenwatch	fastq	S	none
ERR4695940	4.3.1.2.1	India	Pathogenwatch	fastq	S	none
ERR4784656	4.3.1.2.1.1	India	Argimon_et_al	fastq	R: blaSHV-12	IncX3
ERR4785048	4.3.1.2.1	India	Pathogenwatch	fastq	S	none
ERR4790663	4.3.1.2	India	Pathogenwatch	fastq	S	none
ERR4790666	4.3.1.2	India	Pathogenwatch	fastq	S	none
ERR4790719	4.3.1.2	India	Pathogenwatch	fastq	S	none
ERR4790757	4.3.1.2.1	India	Pathogenwatch	fastq	S	none
ERR4790763	4.3.1.2	India	Pathogenwatch	fastq	S	IncFIB
ERR4790791	4.3.1.2.1	India	Pathogenwatch	fastq	S	none
ERR4790804	4.3.1.2	India	Pathogenwatch	fastq	S	none
ERR4790808	4.3.1.2.1	India	Pathogenwatch	fastq	S	none
ERR4992676	4.3.1.2.1	India	Pathogenwatch	fastq	S	none
ERR4992694	4.3.1.2	India	Pathogenwatch	fastq	S	none
ERR4992713	4.3.1.2	India	Pathogenwatch	fastq	S	none
ERR4992744	4.3.1.2.1	India	Pathogenwatch	fastq	S	none

Accession	Genotype	Country	Study	Format	Ceftriaxone_res	Plasmids
ERR4992751	4.3.1.2.1	India	Pathogenwatch	fastq	S	none
ERR4992822	4.3.1.2	India	Pathogenwatch	fastq	S	none
ERR5161622	4.3.1.2	Nepal	Pathogenwatch	fastq	S	none
ERR5161625	4.3.1.2	Nepal	Pathogenwatch	fastq	S	none
ERR5161633	4.3.1.2	Nepal	Pathogenwatch	fastq	S	none
ERR5161659	4.3.1.2	Nepal	Pathogenwatch	fastq	S	none
ERR5161705	4.3.1.2	Nepal	Pathogenwatch	fastq	S	none
ERR5161726	4.3.1.2	Nepal	Pathogenwatch	fastq	S	none
ERR5161760	4.3.1.2	Nepal	Pathogenwatch	fastq	S	none
ERR5161772	4.3.1.2	Nepal	Pathogenwatch	fastq	S	none
ERR5161793	4.3.1.2	Nepal	Pathogenwatch	fastq	S	none
ERR5161936	4.3.1.2	Nepal	Pathogenwatch	fastq	S	none
ERR5200890	4.3.1.2.1	India	Pathogenwatch	fastq	S	none
ERR5200934	4.3.1.2.1	India	Pathogenwatch	fastq	S	none
ERR5200941	4.3.1.2	India	Pathogenwatch	fastq	S	IncFIB
ERR5200949	4.3.1.2	India	Pathogenwatch	fastq	S	none
ERR5200986	4.3.1.2	India	Pathogenwatch	fastq	S	none
ERR5201001	4.3.1.2	India	Pathogenwatch	fastq	S	none
ERR5201012	4.3.1.2.1	India	Pathogenwatch	fastq	S	none
ERR5201013	4.3.1.2.1	India	Pathogenwatch	fastq	S	none
ERR5201051	4.3.1.2.1	India	Pathogenwatch	fastq	S	none
ERR5201084	4.3.1.2	India	Pathogenwatch	fastq	S	none
ERR5201089	4.3.1.2.1	India	Pathogenwatch	fastq	S	none
ERR5201109	4.3.1.2	India	Pathogenwatch	fastq	S	none
ERR5201115	4.3.1.2.1	India	Pathogenwatch	fastq	S	none
ERR5201120	4.3.1.2.1	India	Pathogenwatch	fastq	S	none
ERR5201163	4.3.1.2	India	Pathogenwatch	fastq	S	IncFIB
ERR5201165	4.3.1.2	India	Pathogenwatch	fastq	S	none
ERR5201206	4.3.1.2	India	Pathogenwatch	fastq	S	none
ERR5201208	4.3.1.2	India	Pathogenwatch	fastq	S	none
ERR5201230	4.3.1.2	India	Pathogenwatch	fastq	S	none
ERR5201291	4.3.1.2	India	Pathogenwatch	fastq	S	none
ERR5201311	4.3.1.2.1	India	Pathogenwatch	fastq	S	none
ERR5201336	4.3.1.2.1	India	Pathogenwatch	fastq	S	none
ERR5201351	4.3.1.2	India	Pathogenwatch	fastq	S	none
ERR5311427	4.3.1.2.1	Nepal	Pathogenwatch	fastq	S	none
ERR5311469	4.3.1.2	Nepal	Pathogenwatch	fastq	S	none
ERR5311488	4.3.1.2.1	Nepal	Pathogenwatch	fastq	S	none
ERR5311499	4.3.1.2	Nepal	Pathogenwatch	fastq	S	none
ERR5311506	4.3.1.2	Nepal	Pathogenwatch	fastq	S	none
ERR5311519	4.3.1.2	Nepal	Pathogenwatch	fastq	S	none
ERR5375896	4.3.1.2	Nepal	Pathogenwatch	fastq	S	none
ERR5375959	4.3.1.2.1	Nepal	Pathogenwatch	fastq	S	none
ERR5375984	4.3.1.2	Nepal	Pathogenwatch	fastq	S	none
ERR5376054	4.3.1.2	Nepal	Pathogenwatch	fastq	S	none
ERR5376065	4.3.1.2	Nepal	Pathogenwatch	fastq	S	none
ERR5376071	4.3.1.2	Nepal	Pathogenwatch	fastq	S	none
ERR5376073	4.3.1.2	Nepal	Pathogenwatch	fastq	S	none
ERR5376082	4.3.1.2	Nepal	Pathogenwatch	fastq	S	none
ERR5376093	4.3.1.2.1	Nepal	Pathogenwatch	fastq	S	none
ERR5376094	4.3.1.2.1	Nepal	Pathogenwatch	fastq	S	none
ERR5376104	4.3.1.2	Nepal	Pathogenwatch	fastq	S	none
ERR5376126	4.3.1.2	Nepal	Pathogenwatch	fastq	S	none
ERR5376135	4.3.1.2	Nepal	Pathogenwatch	fastq	S	none
ERR5376169	4.3.1.2	Nepal	Pathogenwatch	fastq	S	none
ERR5376178	4.3.1.2	Nepal	Pathogenwatch	fastq	S	none
ERR6118082	4.3.1.2	India	Pathogenwatch	fastq	S	none
ERR6118102	4.3.1.2	India	Pathogenwatch	fastq	S	none
ERR6118129	4.3.1.2	India	Pathogenwatch	fastq	S	none
ERR6118152	4.3.1.2.1	India	Pathogenwatch	fastq	S	none
ERR6118155	4.3.1.2	India	Pathogenwatch	fastq	S	none
ERR6118163	4.3.1.2	India	Pathogenwatch	fastq	S	none
ERR6294371	4.3.1.2	Nepal	Pathogenwatch	fastq	S	none
ERR6294387	4.3.1.2.1	Nepal	Pathogenwatch	fastq	S	none
ERR6294417	4.3.1.2	Nepal	Pathogenwatch	fastq	S	none

Accession	Genotype	Country	Study	Format	Ceftriaxone_res	Plasmids
ERR6294418	4.3.1.2	Nepal	Pathogenwatch	fastq	S	none
ERR6294420	4.3.1.2	Nepal	Pathogenwatch	fastq	S	none
ERR6294421	4.3.1.2	Nepal	Pathogenwatch	fastq	S	none
ERR6294427	4.3.1.2.1	Nepal	Pathogenwatch	fastq	S	none
ERR6294435	4.3.1.2	Nepal	Pathogenwatch	fastq	S	none
ERR6294451	4.3.1.2	Nepal	Pathogenwatch	fastq	S	none
ERR6294545	4.3.1.2	Nepal	Pathogenwatch	fastq	S	none
ERR6294587	4.3.1.2	Nepal	Pathogenwatch	fastq	S	none
ERR6294588	4.3.1.2	Nepal	Pathogenwatch	fastq	S	none
ERR6294612	4.3.1.2	Nepal	Pathogenwatch	fastq	S	none
ERR6294629	4.3.1.2.1	Nepal	Pathogenwatch	fastq	S	none
ERR6294637	4.3.1.2	Nepal	Pathogenwatch	fastq	S	none
ERR6294652	4.3.1.2	Nepal	Pathogenwatch	fastq	S	none
ERR6294690	4.3.1.2	Nepal	Pathogenwatch	fastq	S	none
CP052767.1	4.3.1.2.1.1	India	Jacob_et_al	fasta	R: blaSHV-12	IncX3
CP053702.1	4.3.1.2	India	Samajpathi_et_al	fasta	R: blaTEM1D-blaDHA	IncN
GCF_001906805.1	4.3.1.2.1.1	India	Rodrigues_et_al	fasta	R: blaSHV-12	IncX3
GCF_001906765.1	4.3.1.2.1.1	India	Rodrigues_et_al	fasta	R: blaSHV-12	IncX3
GCF_001906785.1	4.3.1.2.1.1	India	Rodrigues_et_al	fasta	R: blaSHV-12	IncX3
ERR4303880	4.3.1.1	Bangladesh	da_Silva_et_al	fastq	S	IncFIB
ERR4362605	4.3.1.1	Bangladesh	da_Silva_et_al	fastq	S	IncFIB
ERR4326164	4.3.1.3.Bdq	Bangladesh	da_Silva_et_al	fastq	S	IncFIB
ERR4304069	4.3.1.3.Bdq	Bangladesh	da_Silva_et_al	fastq	S	IncFIB
ERR4325922	4.3.1.3	Bangladesh	da_Silva_et_al	fastq	S	IncFIAHI1
ERR4326166	4.3.1.3.Bdq	Bangladesh	da_Silva_et_al	fastq	S	IncFIB
ERR4326114	4.3.1.3.Bdq	Bangladesh	da_Silva_et_al	fastq	S	IncFIB
ERR4326162	4.3.1.1	Bangladesh	da_Silva_et_al	fastq	S	IncFIB
ERR4325952	4.3.1.3.Bdq	Bangladesh	da_Silva_et_al	fastq	S	IncFIB
ERR4325915	4.3.1.3.Bdq	Bangladesh	da_Silva_et_al	fastq	S	IncFIB
ERR4326105	4.3.1.1	Bangladesh	da_Silva_et_al	fastq	S	IncFIB
ERR4325844	4.3.1.1	Bangladesh	da_Silva_et_al	fastq	S	IncFIB
ERR4326107	4.3.1.1	Bangladesh	da_Silva_et_al	fastq	S	IncFIB
ERR4303916	4.3.1.1	Bangladesh	da_Silva_et_al	fastq	S	IncFIB
ERR4303945	4.3.1.1	Bangladesh	da_Silva_et_al	fastq	S	IncFIB
ERR4362609	4.3.1.3.Bdq	Bangladesh	da_Silva_et_al	fastq	S	IncFIB
ERR4362467	4.3.1.3	Bangladesh	da_Silva_et_al	fastq	S	none
ERR4362455	4.3.1.1	Bangladesh	da_Silva_et_al	fastq	S	IncFIB
ERR4325931	4.3.1.1	Bangladesh	da_Silva_et_al	fastq	S	IncFIB
ERR4325833	4.3.1.3.Bdq	Bangladesh	da_Silva_et_al	fastq	S	IncFIB
ERR4326143	4.3.1.3.Bdq	Bangladesh	da_Silva_et_al	fastq	S	IncFIB
ERR4326099	4.3.1.3.Bdq	Bangladesh	da_Silva_et_al	fastq	S	IncFIB
ERR4326147	4.3.1	Bangladesh	da_Silva_et_al	fastq	S	none
ERR4326051	4.3.1.1	Bangladesh	da_Silva_et_al	fastq	S	IncFIB
ERR4362341	4.3.1.3	Bangladesh	da_Silva_et_al	fastq	S	none
ERR4362573	4.3.1.3	Bangladesh	da_Silva_et_al	fastq	S	none
ERR4303855	4.3.1.3	Bangladesh	da_Silva_et_al	fastq	S	none
ERR4326074	4.3.1.3.Bdq	Bangladesh	da_Silva_et_al	fastq	S	IncFIB
ERR4326049	4.3.1.1	Bangladesh	da_Silva_et_al	fastq	S	IncFIB
ERR4326119	4.3.1.3.Bdq	Bangladesh	da_Silva_et_al	fastq	S	IncFIB
ERR4326020	4.3.1.3.Bdq	Bangladesh	da_Silva_et_al	fastq	S	IncFIB
ERR4362683	4.3.1.1	Bangladesh	da_Silva_et_al	fastq	S	IncFIB
ERR4362679	4.3.1.1	Bangladesh	da_Silva_et_al	fastq	S	none
ERR4303935	4.3.1.1	Bangladesh	da_Silva_et_al	fastq	S	IncFIB
ERR4362427	4.3.1.1	Bangladesh	da_Silva_et_al	fastq	S	none
ERR4362435	4.3.1.1	Bangladesh	da_Silva_et_al	fastq	S	IncFIB
ERR4303847	4.3.1.3	Bangladesh	da_Silva_et_al	fastq	S	none
ERR4325971	4.3.1.3	Bangladesh	da_Silva_et_al	fastq	S	none
ERR4326121	4.3.1.1	Bangladesh	da_Silva_et_al	fastq	S	IncFIB
ERR4326038	4.3.1.1	Bangladesh	da_Silva_et_al	fastq	S	IncFIB
ERR4325845	4.3.1.1	Bangladesh	da_Silva_et_al	fastq	S	IncFIB
ERR4326165	4.3.1.3.Bdq	Bangladesh	da_Silva_et_al	fastq	S	IncFIB
ERR4326177	4.3.1.1	Bangladesh	da_Silva_et_al	fastq	S	none
ERR4326131	4.3.1.1	Bangladesh	da_Silva_et_al	fastq	S	none
ERR4303734	4.3.1.3.Bdq	Bangladesh	da_Silva_et_al	fastq	S	IncFIB

Accession	Genotype	Country	Study	Format	Ceftriaxone_res	Plasmids
ERR4326027	4.3.1.3.Bdq	Bangladesh	da_Silva_et_al	fastq	S	IncFIB
ERR4326015	4.3.1.1	Bangladesh	da_Silva_et_al	fastq	S	none
ERR4326124	4.3.1.1	Bangladesh	da_Silva_et_al	fastq	S	none
ERR4326100	4.3.1.1	Bangladesh	da_Silva_et_al	fastq	S	IncFIB
ERR4326065	4.3.1.1	Bangladesh	da_Silva_et_al	fastq	S	IncFIB
ERR4325953	4.3.1.3.Bdq	Bangladesh	da_Silva_et_al	fastq	S	IncFIB
ERR4326079	4.3.1.3.Bdq	Bangladesh	da_Silva_et_al	fastq	S	IncFIB
ERR4325956	4.3.1.3.Bdq	Bangladesh	da_Silva_et_al	fastq	S	IncFIB
ERR4362539	4.3.1.3	Bangladesh	da_Silva_et_al	fastq	S	none
ERR4362430	4.3.1.3.Bdq	Bangladesh	da_Silva_et_al	fastq	S	IncFIB
ERR4362369	4.3.1.3.Bdq	Bangladesh	da_Silva_et_al	fastq	S	IncFIB
ERR4362381	4.3.1.3.Bdq	Bangladesh	da_Silva_et_al	fastq	S	IncFIB
ERR4362348	4.3.1.1	Bangladesh	da_Silva_et_al	fastq	S	IncFIB
ERR4362603	4.3.1.3.Bdq	Bangladesh	da_Silva_et_al	fastq	S	IncFIB
ERR4362421	4.3.1.3.Bdq	Bangladesh	da_Silva_et_al	fastq	S	IncFIB
ERR4362368	4.3.1.1	Bangladesh	da_Silva_et_al	fastq	S	IncFIB
ERR4362392	4.3.1.3.Bdq	Bangladesh	da_Silva_et_al	fastq	S	IncFIB
ERR4362376	4.3.1.3.Bdq	Bangladesh	da_Silva_et_al	fastq	S	IncFIB
ERR4303998	4.3.1.3	Bangladesh	da_Silva_et_al	fastq	S	none
ERR4303748	4.3.1.1	Bangladesh	da_Silva_et_al	fastq	S	IncFIB
ERR4304070	4.3.1.3	Bangladesh	da_Silva_et_al	fastq	S	none
ERR4304078	4.3.1.3.Bdq	Bangladesh	da_Silva_et_al	fastq	S	IncFIB
ERR4362551	4.3.1.1	Bangladesh	da_Silva_et_al	fastq	S	IncFIB
ERR4362442	4.3.1.3	Bangladesh	da_Silva_et_al	fastq	S	none
ERR4362342	4.3.1.1	Bangladesh	da_Silva_et_al	fastq	S	IncFIB
ERR4325840	4.3.1.1	Bangladesh	da_Silva_et_al	fastq	S	IncFIB
ERR4304106	4.3.1.1	Bangladesh	da_Silva_et_al	fastq	S	IncFIB
ERR4362418	4.3.1.3.Bdq	Bangladesh	da_Silva_et_al	fastq	S	IncFIB
ERR4362336	4.3.1.1	Bangladesh	da_Silva_et_al	fastq	S	IncFIB
ERR4362583	4.3.1.1	Bangladesh	da_Silva_et_al	fastq	S	IncFIB
ERR4362587	4.3.1.3.Bdq	Bangladesh	da_Silva_et_al	fastq	S	IncFIB
ERR4362372	4.3.1.3.Bdq	Bangladesh	da_Silva_et_al	fastq	S	IncFIB
ERR4303942	4.3.1.3.Bdq	Bangladesh	da_Silva_et_al	fastq	S	IncFIB
ERR4303861	4.3.1.1	Bangladesh	da_Silva_et_al	fastq	S	IncFIB
ERR4303740	4.3.1.3	Bangladesh	da_Silva_et_al	fastq	S	none
ERR4303772	4.3.1.3.Bdq	Bangladesh	da_Silva_et_al	fastq	S	IncFIB
ERR4303800	4.3.1.3.Bdq	Bangladesh	da_Silva_et_al	fastq	S	IncFIB
ERR4304094	4.3.1	Bangladesh	da_Silva_et_al	fastq	S	none
ERR4362708	4.3.1.2	Bangladesh	da_Silva_et_al	fastq	S	none
ERR4362512	4.3.1.1	Bangladesh	da_Silva_et_al	fastq	S	IncFIB
ERR4362338	4.3.1.1	Bangladesh	da_Silva_et_al	fastq	S	IncFIB
ERR4362337	4.3.1.1	Bangladesh	da_Silva_et_al	fastq	S	none
ERR4362556	4.3.1.2	Bangladesh	da_Silva_et_al	fastq	S	none
ERR4362694	4.3.1.1	Bangladesh	da_Silva_et_al	fastq	S	IncFIB
ERR4362660	4.3.1.1	Bangladesh	da_Silva_et_al	fastq	S	none
ERR4362677	4.3.1.2	Bangladesh	da_Silva_et_al	fastq	S	none
ERR4362596	4.3.1.1	Bangladesh	da_Silva_et_al	fastq	S	IncFIB
ERR4362632	4.3.1.1	Bangladesh	da_Silva_et_al	fastq	S	none
ERR4362698	4.3.1.1	Bangladesh	da_Silva_et_al	fastq	S	IncFIB
ERR4362689	4.3.1.1	Bangladesh	da_Silva_et_al	fastq	S	IncFIB
ERR4362640	4.3.1.1	Bangladesh	da_Silva_et_al	fastq	S	IncFIB
ERR4362669	4.3.1.1	Bangladesh	da_Silva_et_al	fastq	S	IncFIB
ERR4362681	4.3.1.1	Bangladesh	da_Silva_et_al	fastq	S	IncFIB
ERR4362637	4.3.1.1	Bangladesh	da_Silva_et_al	fastq	S	IncFIB
ERR4303899	4.3.1.1	Bangladesh	da_Silva_et_al	fastq	S	none
ERR4362508	4.3.1.1	Bangladesh	da_Silva_et_al	fastq	S	IncFIB
ERR4303985	4.3.1.1	Bangladesh	da_Silva_et_al	fastq	S	none
ERR4304005	4.3.1.1	Bangladesh	da_Silva_et_al	fastq	S	IncFIB
ERR4303972	4.3.1.3.Bdq	Bangladesh	da_Silva_et_al	fastq	S	IncFIB
SRR10209615	4.3.1.2.1	India	Pathogenwatch	fastq	S	none
SRR10222050	4.3.1.2.1	Pakistan	Pathogenwatch	fastq	S	none
SRR10504947	4.3.1.2	India	Pathogenwatch	fastq	S	none
SRR10593982	4.3.1.2	India	Pathogenwatch	fastq	S	IncFIB
SRR10673983	4.3.1.2	India	Pathogenwatch	fastq	S	none
SRR10738020	4.3.1.2	India	Pathogenwatch	fastq	S	none

Accession	Genotype	Country	Study	Format	Ceftriaxone_res	Plasmids
SRR10814052	4.3.1.2.1	India	Pathogenwatch	fastq	S	none
SRR10882155	4.3.1.2	India	Pathogenwatch	fastq	S	none
SRR10993298	4.3.1.2	India	Pathogenwatch	fastq	S	none
SRR12278699	4.3.1.2.EA3	Rwanda	Pathogenwatch	fastq	S	none
SRR12278703	4.3.1.2.EA3	Rwanda	Pathogenwatch	fastq	S	none
SRR12278714	4.3.1.2.EA3	Rwanda	Pathogenwatch	fastq	S	IncHI1
SRR12567842	4.3.1.2.1	India	Pathogenwatch	fastq	S	none
SRR13246080	4.3.1.2.1	India	Pathogenwatch	fastq	S	none
SRR13246424	4.3.1.2	India	Pathogenwatch	fastq	S	none
SRR14864523	4.3.1.2.1	India	Pathogenwatch	fastq	S	none
SRR14864567	4.3.1.2	India	Pathogenwatch	fastq	S	IncFIB
SRR15146277	4.3.1.2	India	Pathogenwatch	fastq	S	none
SRR1957928	4.3.1.2.1	India	Pathogenwatch	fastq	S	none
SRR1967393	4.3.1.2	India	Pathogenwatch	fastq	S	none
SRR3048761	4.3.1.2	India	Pathogenwatch	fastq	S	none
SRR3048864	4.3.1.2	United Kingdom	Pathogenwatch	fastq	S	none
SRR3049299	4.3.1.2.EA3	Uganda	Pathogenwatch	fastq	S	none
SRR5361122	4.3.1.2.1	India	Pathogenwatch	fastq	S	none
SRR5500459	4.3.1.2	India	Pathogenwatch	fastq	S	none
SRR5500479	4.3.1.2	United Kingdom	Pathogenwatch	fastq	S	none
SRR5500496	4.3.1.2	India	Pathogenwatch	fastq	S	none
SRR5500523	4.3.1.2.1	India	Pathogenwatch	fastq	S	none
SRR5633086	4.3.1.2	India	Pathogenwatch	fastq	S	none
SRR5974914	4.3.1.2	India	Pathogenwatch	fastq	S	none
SRR5986536	4.3.1.2.1	India	Pathogenwatch	fastq	S	none
SRR5989303	4.3.1.2	India	Pathogenwatch	fastq	S	none
SRR5989310	4.3.1.2	India	Pathogenwatch	fastq	S	IncN
SRR6354013	4.3.1.2	India	Pathogenwatch	fastq	S	none
SRR7049884	4.3.1.2.1.1	India	Pathogenwatch	fastq	S	none
SRR7165016	4.3.1.2	India	Pathogenwatch	fastq	S	none
SRR7165414	4.3.1.2	United Kingdom	Pathogenwatch	fastq	S	none
SRR7165449	4.3.1.2.1	India	Pathogenwatch	fastq	S	none
SRR7165510	4.3.1.2	India	Pathogenwatch	fastq	S	none
SRR7165566	4.3.1.2.1	India	Pathogenwatch	fastq	S	none
SRR7165579	4.3.1.2	India	Pathogenwatch	fastq	S	none
SRR7165745	4.3.1.2	India	Pathogenwatch	fastq	S	none
SRR7170403	4.3.1.2.1	India	Pathogenwatch	fastq	S	none
SRR7300447	4.3.1.2	India	Pathogenwatch	fastq	S	none
SRR7469055	4.3.1.2.1	India	Pathogenwatch	fastq	S	none
SRR7469166	4.3.1.2.1	India	Pathogenwatch	fastq	S	none
SRR7495548	4.3.1.2.1	India	Pathogenwatch	fastq	S	none
SRR8117623	4.3.1.2.1	Myanmar	Pathogenwatch	fastq	S	none
SRR8165230	4.3.1.2	India	Pathogenwatch	fastq	S	none
SRR8283327	4.3.1.2	India	Pathogenwatch	fastq	S	none
SRR8490821	4.3.1.2.1	Pakistan	Pathogenwatch	fastq	S	none
SRR8498996	4.3.1.2.1	India	Pathogenwatch	fastq	S	none
SRR8509417	4.3.1.2.1	India	Pathogenwatch	fastq	S	none
SRR8514765	4.3.1.2.1.1	India	Pathogenwatch	fastq	R: blaSHV-12	IncX3
SRR8786836	4.3.1.2	India	Pathogenwatch	fastq	S	none
SRR9261535	4.3.1.2.1	Pakistan	Pathogenwatch	fastq	S	none
SRR9283165	4.3.1.2	India	Pathogenwatch	fastq	S	IncFIB
SRR9616298	4.3.1.2.1	India	Pathogenwatch	fastq	S	none
ERR14100491	4.3.1.1	Bangladesh	This_Study	fastq	S	IncFIB
ERR14100639	4.3.1.1	Bangladesh	This_Study	fastq	S	IncFIB
ERR14100642	4.3.1.1	Bangladesh	This_Study	fastq	S	IncFIB
ERR14100643	4.3.1.1	Bangladesh	This_Study	fastq	S	IncFIB
ERR14100645	4.3.1.1	Bangladesh	This_Study	fastq	S	IncFIB
ERR14100646	4.3.1.1	Bangladesh	This_Study	fastq	S	IncFIB
ERR14101690	4.3.1.3.Bdq	Bangladesh	This_Study	fastq	S	IncFIB
ERR14100722	4.3.1.1	Bangladesh	This_Study	fastq	S	IncFIB
ERR14100723	4.3.1.1	Bangladesh	This_Study	fastq	S	none
ERR14100724	4.3.1.1	Bangladesh	This_Study	fastq	S	IncFIB

Accession	Genotype	Country	Study	Format	Ceftriaxone_res	Plasmids
ERR14100725	4.3.1.1	Bangladesh	This_Study	fastq	S	none
ERR14100726	4.3.1.1	Bangladesh	This_Study	fastq	S	IncFIB
ERR14100727	4.3.1.3.Bdq	Bangladesh	This_Study	fastq	S	IncFIB
ERR14100728	4.3.1.1	Bangladesh	This_Study	fastq	S	none
ERR14100729	4.3.1.1	Bangladesh	This_Study	fastq	S	none
ERR14100730	4.3.1.1	Bangladesh	This_Study	fastq	S	none
ERR14100731	4.3.1.1	Bangladesh	This_Study	fastq	S	IncFIB
ERR14100732	4.3.1.1	Bangladesh	This_Study	fastq	S	IncFIB
ERR14100733	4.3.1.1	Bangladesh	This_Study	fastq	S	IncFIB
ERR14100734	4.3.1.1	Bangladesh	This_Study	fastq	S	IncFIB
ERR14100735	4.3.1.1	Bangladesh	This_Study	fastq	S	IncFIB
ERR14100736	4.3.1.3.Bdq	Bangladesh	This_Study	fastq	S	IncFIB
ERR14100737	4.3.1.2	Bangladesh	This_Study	fastq	S	IncFIB
ERR14100739	4.3.1.1	Bangladesh	This_Study	fastq	S	IncFIB
ERR14100740	4.3.1.2	Bangladesh	This_Study	fastq	S	none
ERR14100742	4.3.1.1	Bangladesh	This_Study	fastq	S	IncFIB
ERR14100743	4.3.1.1	Bangladesh	This_Study	fastq	S	none
ERR14100744	4.3.1.1	Bangladesh	This_Study	fastq	S	none
ERR14100745	4.3.1.1	Bangladesh	This_Study	fastq	S	IncFIB
ERR14100746	4.3.1.1	Bangladesh	This_Study	fastq	S	none
ERR14101136	4.3.1.1	Bangladesh	This_Study	fastq	S	none
ERR14101137	4.3.1.1	Bangladesh	This_Study	fastq	S	IncFIB
ERR14101138	4.3.1.1	Bangladesh	This_Study	fastq	S	none
ERR14101139	4.3.1.1	Bangladesh	This_Study	fastq	S	IncFIB
ERR14101140	4.3.1.1	Bangladesh	This_Study	fastq	S	IncFIB
ERR14101143	4.3.1.2	Bangladesh	This_Study	fastq	R: blaCTX-M-15	IncY
ERR14101145	4.3.1.2	Bangladesh	This_Study	fastq	R: blaCTX-M-15	IncY
ERR14101146	4.3.1.2	Bangladesh	This_Study	fastq	R: blaCTX-M-15	IncY
ERR14101224	4.3.1.2	Bangladesh	This_Study	fastq	R: blaCTX-M-15	IncY
ERR14101225	4.3.1.2	Bangladesh	This_Study	fastq	R: blaCTX-M-15	IncY
ERR14101226	4.3.1.2	Bangladesh	This_Study	fastq	R: blaCTX-M-15	IncY
ERR14101227	4.3.1.2	Bangladesh	This_Study	fastq	R: blaCTX-M-15	IncY
ERR14101228	4.3.1.2	Bangladesh	This_Study	fastq	R: blaCTX-M-15	IncY
ERR14101229	4.3.1.2	Bangladesh	This_Study	fastq	R: blaCTX-M-15	IncY
ERR14101231	4.3.1.2	Bangladesh	This_Study	fastq	R: blaCTX-M-15	IncY
ERR14101234	4.3.1.2	Bangladesh	This_Study	fastq	R: blaCTX-M-15	IncY
ERR14101278	4.3.1.2	Bangladesh	This_Study	fastq	R: blaCTX-M-15	IncY
ERR14101279	4.3.1.2	Bangladesh	This_Study	fastq	R: blaCTX-M-15	IncY
ERR14101280	4.3.1.1	Bangladesh	This_Study	fastq	S	IncFIB
ERR14101282	4.3.1.2	Bangladesh	This_Study	fastq	R: blaCTX-M-15	IncY
ERR14101283	4.3.1.2	Bangladesh	This_Study	fastq	R: blaCTX-M-15	IncY
ERR14101672	4.3.1.2	Bangladesh	This_Study	fastq	R: blaCTX-M-15	IncY
ERR14101673	4.3.1.2	Bangladesh	This_Study	fastq	R: blaCTX-M-15	IncY

References

1. Thirumoorthy TP, Jacob JJ, Velmurugan A, Teekaraman MP, Shah B, Iyer V, et al. Recent emergence of cephalosporin-resistant *Salmonella* Typhi in India due to the endemic clone acquiring IncFIB(K) plasmid encoding *bla*$_{\text{CTX-M-15}}$ gene. Microbiol Spectr. 2025;13:e0087524. PubMed https://doi.org/10.1128/spectrum.00875-24

2. Sah R, Donovan S, Seth-Smith HMB, Bloemberg G, Wüthrich D, Stephan R, et al. A novel lineage of ceftriaxone-resistant *Salmonella* Typhi from India that is closely related to XDR *S.* Typhi found in Pakistan. Clin Infect Dis. 2020;71:1327–30. PubMed https://doi.org/10.1093/cid/ciz1204

3. Argimón S, Nagaraj G, Shamanna V, Sravani D, Vasanth AK, Prasanna A, et al. Circulation of third-generation cephalosporin resistant *Salmonella* Typhi in Mumbai, India. Clin Infect Dis. 2022;74:2234–7. PubMed https://doi.org/10.1093/cid/ciab897

4. Jacob JJ, Pragasam AK, Vasudevan K, Veeraraghavan B, Kang G, John J, et al. *Salmonella* Typhi acquires diverse plasmids from other Enterobacteriaceae to develop cephalosporin resistance. Genomics. 2021;113:2171–6. PubMed https://doi.org/10.1016/j.ygeno.2021.05.003

5. Samajpati S, Pragasam AK, Mandal S, Balaji V, Dutta S. Emergence of ceftriaxone resistant *Salmonella enterica* serovar Typhi in Eastern India. Infect Genet Evol. 2021;96:105093. PubMed https://doi.org/10.1016/j.meegid.2021.105093

6. Rodrigues C, Kapil A, Sharma A, Devanga Ragupathi NK, Inbanathan FY, Veeraraghavan B, et al. Whole-genome shotgun sequencing of cephalosporin-resistant *Salmonella enterica* serovar Typhi. Genome Announc. 2017;5:e01639–16. PubMed https://doi.org/10.1128/genomeA.01639-16

7. da Silva KE, Tanmoy AM, Pragasam AK, Iqbal J, Sajib MSI, Mutreja A, et al. The international and intercontinental spread and expansion of antimicrobial-resistant *Salmonella* Typhi: a genomic epidemiology study. Lancet Microbe. 2022;3:e567–77. PubMed https://doi.org/10.1016/S2666-5247(22)00093-3
